# Heart Rate Influences the Relationship Between Heart Rate Variability and Ventricular Repolarization in Children: A 24-h Holter Study

**DOI:** 10.3390/jcdd13080374

**Published:** 2026-08-07

**Authors:** Seyma Kayali

**Affiliations:** Division of Pediatric Cardiology, Department of Pediatrics, Karabuk University Faculty of Medicine, Karabuk 78050, Türkiye; seymakayali@gmail.com

**Keywords:** heart rate variability, ventricular repolarization, heart rate, pediatric cardiology, Holter monitoring

## Abstract

Background/Objectives: Heart rate variability (HRV) and ventricular repolarization are key noninvasive markers of cardiac autonomic regulation and electrical stability. Their physiological relationship in healthy pediatric populations remains incompletely understood. This study evaluated the association between HRV parameters and ventricular repolarization indices and established age-specific reference values for HRV. Methods: This cross-sectional study included 254 healthy children (5–18 years) who underwent 24 h Holter monitoring. HRV parameters (SDNN, SDANN, RMSSD, pNN50, LF, HF, LF/HF ratio) and repolarization indices (QT, QTc, QTd, QTcd) were analyzed. Age-stratified percentile distributions were generated, and multivariable analyses were performed, adjusting for age, sex, and heart rate. Results: All HRV parameters increased significantly with age (*p* < 0.001), accompanied by a decrease in heart rate. In unadjusted analyses, the QT interval showed positive correlations with HRV indices, particularly SDNN (r = 0.434, *p* < 0.001). However, these associations were no longer significant after adjustment. QTd remained modestly associated with SDNN, whereas QTc showed no association with HRV; sex was the only independent predictor of QTc. Conclusion: In healthy children, the apparent association between HRV and the QT interval appears to be largely explained by heart rate rather than by an independent physiological relationship. These findings highlight the importance of considering heart rate when interpreting associations between autonomic and ventricular repolarization parameters in pediatric studies. Age-specific HRV reference values may support the clinical interpretation of pediatric autonomic and electrophysiological assessments.

## 1. Introduction

Heart rate variability (HRV) is a well-established, noninvasive marker reflecting the complex autonomic regulation of the cardiovascular system. It provides critical insights into the dynamic balance between sympathetic and parasympathetic activities, where a higher HRV generally signifies a healthy, adaptive autonomic nervous system, while reduced HRV has been consistently linked to increased cardiovascular risk, arrhythmogenesis, and adverse outcomes across various clinical populations [1,2]. HRV parameters derived from 24 h ambulatory electrocardiographic (Holter) recordings are widely regarded as the gold standard for evaluating cardiac autonomic function, offering a comprehensive assessment of autonomic modulation during daily activities and sleep [3,4].

Ventricular repolarization, a fundamental phase of the cardiac cycle, is represented by electrocardiographic indices such as the QT interval, corrected QT interval (QTc), QT dispersion (QTd), and corrected QT dispersion (QTcd). These parameters provide essential information regarding the duration and spatial heterogeneity of myocardial recovery [5]. Increased dispersion of repolarization, in particular, is considered a marker of electrical instability and has been strongly associated with an increased susceptibility to life-threatening ventricular arrhythmias and sudden cardiac death [6,7]. Consequently, these repolarization markers have become indispensable tools for arrhythmic risk stratification in both adult and pediatric cardiology [8].

The autonomic nervous system plays a pivotal role in modulating ventricular electrophysiology. Sympathetic and parasympathetic influences can significantly alter myocardial repolarization properties, thereby affecting QT interval dynamics and repolarization heterogeneity [9]. While sympathetic stimulation typically shortens the action potential duration and QT interval, parasympathetic activity tends to prolong repolarization and enhance its stability [10]. Several studies in adult populations have demonstrated a significant association between HRV parameters and ventricular repolarization indices, suggesting a profound interaction between autonomic modulation and myocardial electrical stability [11,12]. However, the precise nature of this relationship in the pediatric population remains an area of active investigation.

Data regarding the relationship between HRV and ventricular repolarization parameters in children and adolescents are currently limited. Previous research in this field has predominantly focused on specific patient groups, such as those with obesity, metabolic syndrome, or congenital heart disease, where autonomic dysfunction is often a confounding factor [13,14]. Since cardiac autonomic regulation and ventricular electrophysiology undergo significant maturational changes from childhood through adolescence, establishing the relationship between these parameters in healthy individuals is crucial [15,16]. Although several studies have examined HRV or ventricular repolarization parameters separately, data evaluating the association between autonomic modulation and repolarization heterogeneity using 24 h Holter recordings in healthy pediatric populations remain scarce. Furthermore, there is a pressing clinical need for age-stratified normative reference values for HRV parameters to accurately interpret autonomic function in the pediatric age group [17]. Recent meta-analytic evidence has attempted to establish age-specific reference ranges for HRV parameters in pediatric populations based on pooled data from Holter recordings. However, these studies do not address individual-level physiological interactions between autonomic modulation and ventricular repolarization [18]. A critical methodological challenge in this field is the strong dependence of both HRV and the QT interval on heart rate. Failure to adequately account for this shared dependency may lead to spurious associations and misinterpretation of physiological interactions. Despite this, many studies have not rigorously addressed heart rate as a confounding factor, particularly in pediatric populations.

Therefore, the present study aimed to evaluate the relationship between HRV parameters and ventricular repolarization indices (QT, QTc, QTd, and QTcd) obtained from 24 h Holter recordings in a cohort of healthy children. Given that both HRV and ventricular repolarization parameters are influenced by heart rate, a key objective was to determine whether the observed associations persisted after adjustment for heart rate. Additionally, age-specific normative reference values for HRV parameters were established in the pediatric population.

## 2. Methods

### 2.1. Study Design and Population

This cross-sectional observational study was conducted at the Pediatric Cardiology Department of Karabuk University Karabuk Training and Research Hospital between June 2025 and March 2026. The study protocol was approved by the Institutional Ethics Committee of the Karabuk University Faculty of Medicine (Approval No: 2026/2805) and was conducted in accordance with the ethical standards of the Declaration of Helsinki. Written informed consent was obtained from the parents or legal guardians of all participants.

The study population initially comprised children referred to the pediatric cardiology outpatient clinic for routine evaluation or symptoms such as palpitations and chest pain. Following a comprehensive clinical assessment—including physical examination, standard 12-lead electrocardiography (ECG), transthoracic echocardiography, and 24 h Holter monitoring—254 children (aged 5–18 years) with no evidence of cardiovascular or systemic disease were included in the final analysis. Participants were excluded if they had: (i) congenital or acquired heart disease; (ii) previously diagnosed arrhythmias; (iii) hypertension or metabolic disorders; (iv) chronic systemic diseases; or (v) were using medications known to affect cardiac conduction or autonomic function.

### 2.2. Holter ECG Recording and Data Processing

All participants underwent 24 h ambulatory ECG monitoring using a 12-channel digital Holter system (BI9800TL+, Biomedical Instruments Co., Shenzhen, China). Recordings were obtained during routine daily activities, with participants instructed to avoid excessive physical exertion. To ensure data integrity, only recordings with at least 20 h of analyzable data and fewer than 20% artifacts or ectopic beats were included [3]. Following the monitoring period, all recordings were transferred to a dedicated analysis workstation for analysis. Ventricular repolarization parameters (QT, QTc, QTd, and QTcd) were automatically calculated using the integrated Holter analysis software. The software generated hourly measurements throughout the 24 h recording and provided mean 24 h values, which were used for statistical analyses. These conventional ventricular repolarization parameters were selected because they were consistently available from all 24 h Holter recordings included in this retrospective study. Additional repolarization markers, such as the Tpeak–Tend interval, T-wave morphology, and QT variability index, were not available for retrospective analysis using the integrated Holter analysis system. Recordings were reviewed for adequate signal quality, and automatically generated measurements were visually inspected by a pediatric cardiologist to verify appropriate waveform detection and measurement accuracy.

### 2.3. Heart Rate Variability (HRV) Analysis

HRV parameters were derived from the 24 h Holter recordings in accordance with the international standards established by the Task Force of the European Society of Cardiology and the North American Society of Pacing and Electrophysiology [1]. Time-domain indices were calculated from normal-to-normal (NN) intervals after the exclusion of ectopic beats and artifacts. The following parameters were evaluated:

SDNN (ms): Standard deviation of all NN intervals, reflecting overall autonomic activity and the combined influence of sympathetic and parasympathetic activity over the entire recording period. It is considered a marker of global autonomic regulation.

SDANN (ms): Standard deviation of the averages of NN intervals in all 5 min segments, representing long-term components of HRV and reflecting predominantly slower fluctuations in autonomic tone.

RMSSD (ms): Root mean square of successive differences between adjacent NN intervals, reflecting short-term beat-to-beat variability and parasympathetic (vagal) activity.

pNN50 (%): Percentage of successive NN intervals differing by more than 50 ms, representing short-term variability and vagal modulation similar to RMSSD.

Frequency-domain analysis was performed using the Fast Fourier Transform (FFT). Standard frequency bands implemented in the Holter analysis software were used in accordance with current international recommendations for HRV analysis (1). The integrated Holter analysis software performed spectral analysis at 5 min intervals throughout the recording, and the reported 24 h frequency-domain parameters (LF, HF, and LF/HF ratio) represented the overall values calculated from these sequential analyses.

LF (ms^2^): Low-frequency power (0.04–0.15 Hz), reflecting both sympathetic and parasympathetic influences and associated with baroreflex-mediated regulation of blood pressure.

HF (ms^2^): High-frequency power (0.15–0.40 Hz), representing parasympathetic activity and closely linked to predominantly parasympathetic modulation.

LF/HF Ratio: An index commonly used to estimate sympathovagal balance, although its physiological interpretation remains controversial and should be interpreted with caution [1,11].

Nonlinear HRV indices were not evaluated because these parameters were not available for retrospective analysis using the integrated Holter analysis system.

### 2.4. Assessment of Ventricular Repolarization Parameters

Ventricular repolarization indices were extracted from the Holter-derived ECG data using the system’s automated analysis software, followed by manual adjudication of the T-wave offset using the tangent method [19]. The following indices were assessed:

QT Interval: Measured from the onset of the QRS complex to the point where the T-wave returned to the isoelectric baseline.

Corrected QT (QTc): Calculated using Bazett’s formula ($QTc=QT/\sqrt{RR}$), which remains the most widely used correction method in pediatric practice despite its known limitations at extreme heart rates [20,21].

QT Dispersion (QTd): Defined as the difference between the maximum and minimum QT intervals across the recorded leads.

Corrected QT Dispersion (QTcd): Calculated as the difference between the maximum and minimum QTc values [6].

A subset of recordings was manually reviewed to ensure measurement consistency. To assess intraobserver reproducibility, a subset of randomly selected recordings (*n* = 25) was reanalyzed by the same investigator after a two-week interval, blinded to the initial measurements.

### 2.5. Statistical Analysis

Statistical analyses were performed using SPSS software version 25.0 (IBM Corp., Armonk, NY, USA). The normality of the data distribution was assessed using the Kolmogorov–Smirnov test. Continuous variables were expressed as mean ± standard deviation (SD) for normally distributed data or median (interquartile range) for non-normally distributed data. Variables with a non-normal distribution were analyzed using appropriate non-parametric tests.

To evaluate unadjusted associations between HRV parameters and ventricular repolarization indices, Pearson’s correlation coefficient was used for normally distributed variables and Spearman’s rank correlation for non-normally distributed variables [22]. Age-stratified percentile distributions (5th–95th percentiles) were generated for HRV parameters to establish normative reference values.

To account for potential confounding effects, multivariable linear regression analyses were performed to assess the independent associations between HRV parameters and ventricular repolarization indices (QT, QTc, and QTd). Separate models were constructed, including age, sex, and heart rate as covariates. Separate models were also used for each HRV parameter to avoid multicollinearity among HRV indices. Standardized beta coefficients (β) with corresponding *p*-values were reported. Multicollinearity was assessed using variance inflation factor (VIF) values. Bonferroni correction was applied to the univariate correlation analyses to account for multiple testing, and the adjusted significance threshold was set at *p* < 0.002. Additionally, partial correlation analyses were conducted to further evaluate the relationships between HRV parameters and the QT interval after controlling for age, sex, and heart rate. A two-tailed *p*-value < 0.05 was considered statistically significant.

## 3. Results

### 3.1. Study Population and Baseline Characteristics

A total of 254 healthy children (145 males, 57.1%; 109 females, 42.9%) with a mean age of 12.51 ± 3.55 years (range: 5–18 years) were included in the final analysis. The mean heart rate (HR) was 82.17 ± 10.77 bpm. Baseline demographic characteristics, heart rate variability (HRV) parameters, and ventricular repolarization indices are summarized in Table 1. Intraobserver reproducibility was assessed in a subset of recordings. Agreement for QT measurements was excellent (ICC = 0.89, 95% CI: 0.81–0.90), while agreement for QT dispersion (QTd) was moderate (ICC = 0.72, 95% CI: 0.60–0.80).

### 3.2. Age-Stratified HRV Percentile Distributions

**Figure 1:**
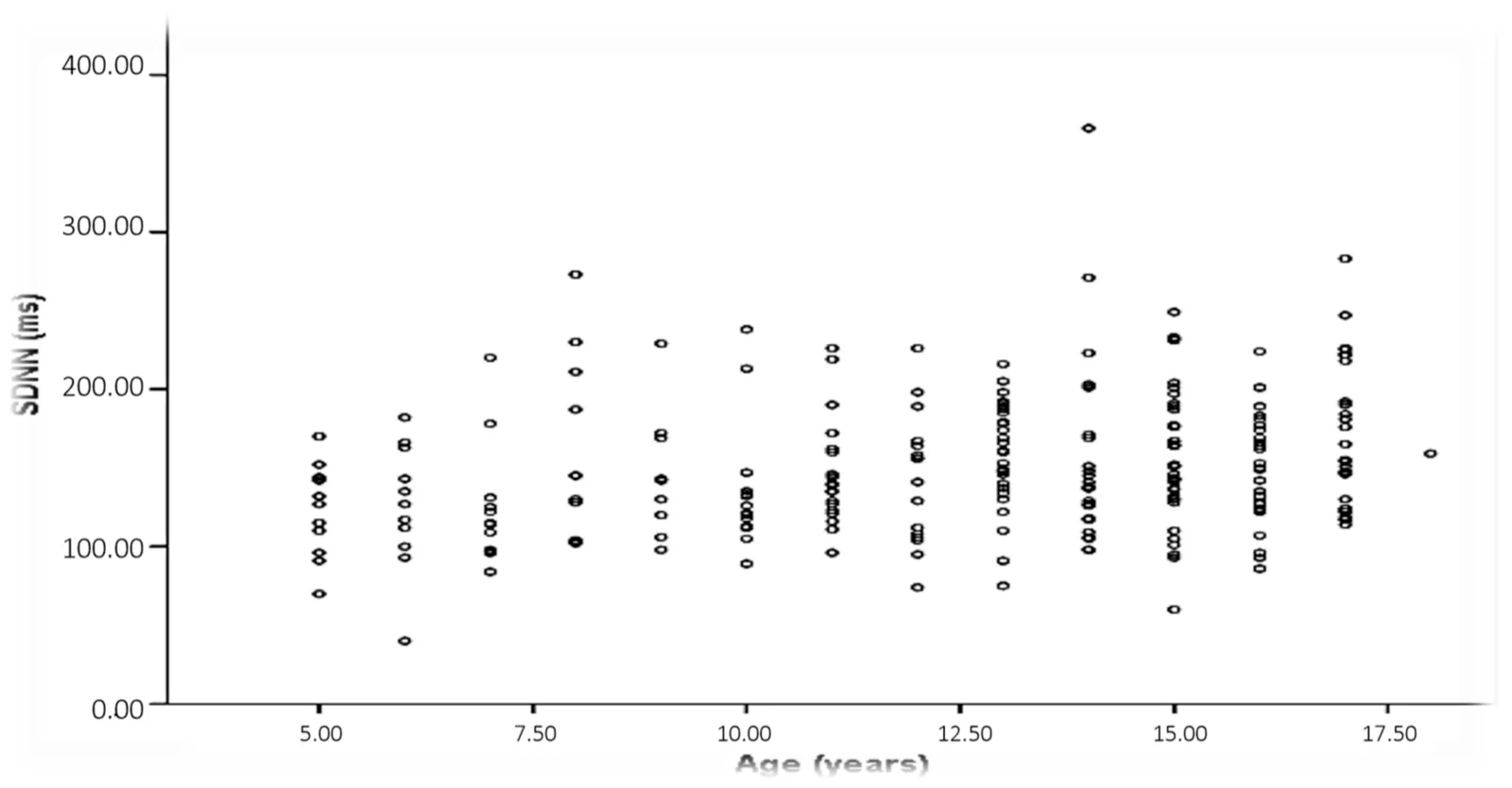
Scatter plot showing the distribution of SDNN values across age in the study population.

HRV parameters were stratified into four age groups (5–8, 9–12, 13–15, and 16–18 years). Age groups were predefined to represent clinically relevant developmental stages during childhood and adolescence. All time-domain and frequency-domain HRV parameters demonstrated significant age-related increases across groups (*p* < 0.001), accompanied by a decline in heart rate (r = −0.471, *p* < 0.001). Age-specific percentile distributions (5th–95th) for HRV indices are presented in Table 2. The distribution of SDNN values across age is shown in Figure 1.

### 3.3. Unadjusted Associations Between HRV and Ventricular Repolarization

**Figure 2:**
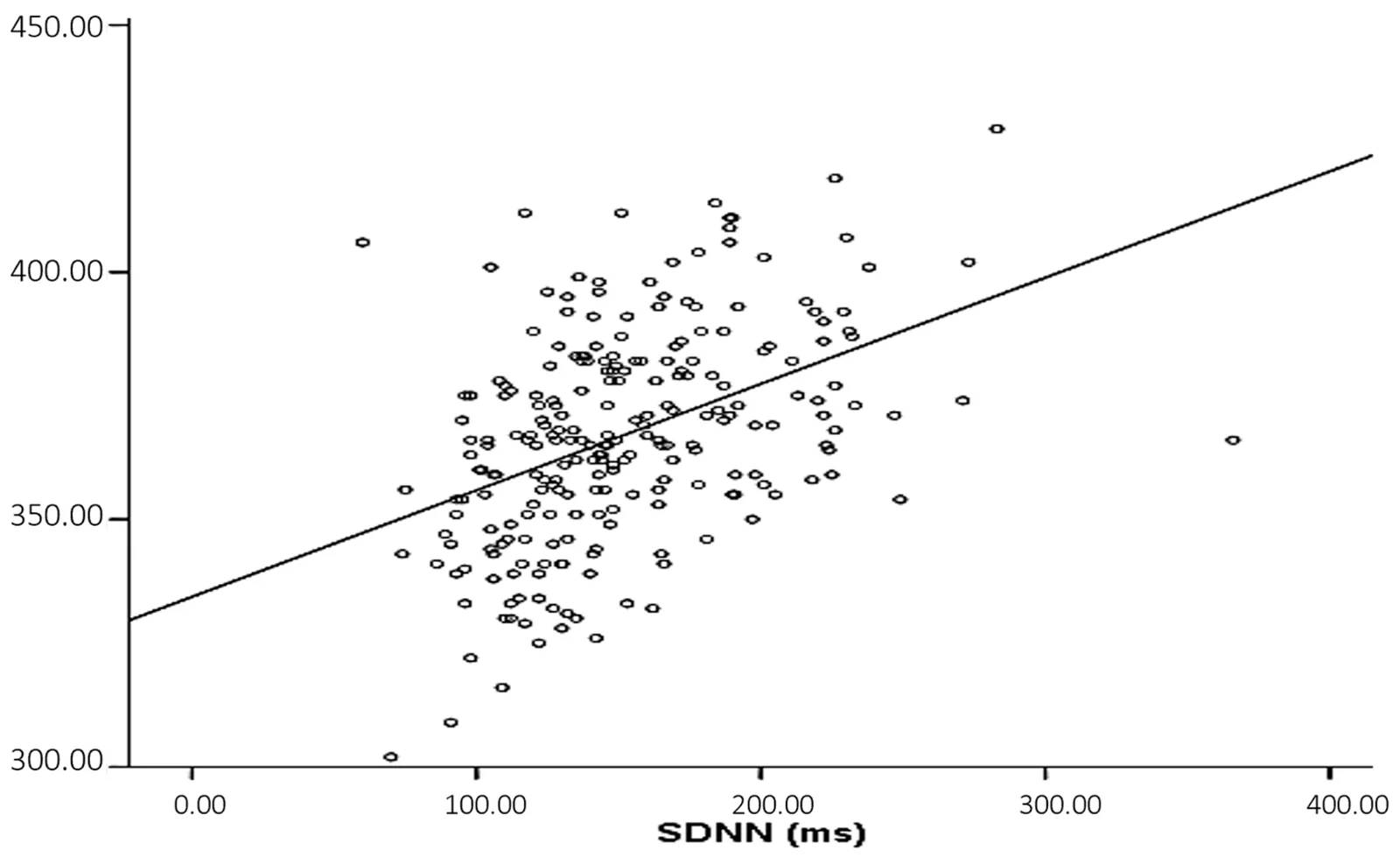
Scatter plot showing the relationship between the QT interval and SDNN.

In unadjusted analyses, the QT interval showed significant positive correlations with all HRV parameters. The strongest associations were observed with SDNN (r = 0.434, *p* < 0.001) and RMSSD (r = 0.426, *p* < 0.001). QT also correlated with SDANN (r = 0.412, *p* < 0.001), pNN50 (r = 0.405, *p* < 0.001), LF (r = 0.437, *p* < 0.001), and HF (r = 0.367, *p* < 0.001). The relationship between theQT interval and SDNN is illustrated in Figure 2.

In contrast, QTc was not significantly correlated with HRV parameters (*p* > 0.05 for all). Repolarization heterogeneity, as measured by QT dispersion (QTd), showed significant positive correlations with SDNN (r = 0.269, *p* < 0.001). Although weaker correlations were observed with RMSSD (r = 0.146, *p* = 0.025) and HF (r = 0.186, *p* = 0.004), these associations did not remain significant after Bonferroni correction. No significant associations were observed between QTcd and HRV indices. Detailed correlation coefficients are presented in Table 3.

### 3.4. Multivariable Analyses Adjusted for Heart Rate, Age, and Sex

To account for potential confounding effects, multivariable linear regression analyses were performed.

After adjustment for heart rate, age, and sex, the previously observed associations between the QT interval and HRV parameters were no longer statistically significant, indicating that these relationships were largely explained by heart rate. Similarly, partial correlation analyses controlling for these variables confirmed the absence of independent associations between QT and HRV indices.

In contrast, SDNN remained modestly associated with QT dispersion (QTd) in adjusted models, whereas RMSSD and other HRV parameters were not independently associated with QTd.

For QTc, multivariable analysis identified sex as the only significant independent predictor (β = 0.294, *p* < 0.001), while age, heart rate, and HRV parameters were not significantly associated. The model explained approximately 11% of the variance in QTc.

### 3.5. Sex-Specific Differences

Females exhibited significantly lower HRV values compared to males, including SDNN (r = −0.272, *p* < 0.001), SDANN (r = −0.245, *p* < 0.001), RMSSD (r = −0.312, *p* < 0.001), and pNN50 (r = −0.288, *p* < 0.001). In addition, females had higher QTc values (r = 0.294, *p* < 0.001). These findings indicate sex-related differences in both autonomic modulation and ventricular repolarization in the pediatric population.

## 4. Discussion

This study evaluated the relationship between heart rate variability (HRV) and ventricular repolarization parameters in a cohort of healthy children and adolescents while also establishing age-stratified reference values for HRV derived from 24 h Holter recordings. The principal finding is that although significant associations were observed between HRV parameters and the QT interval in unadjusted analyses, these associations were largely explained by the shared influence of heart rate. After adjustment for heart rate, age, and sex, most associations were substantially attenuated or no longer significant. Likewise, the absence of independent associations between HRV parameters and QTc supports the interpretation that heart rate is an important confounding factor when evaluating the relationship between autonomic function and ventricular repolarization in healthy children. Consistent with previous pediatric studies, we observed significant age-related increases in both time-domain and frequency-domain HRV parameters, accompanied by a decline in resting heart rate. These findings reflect the progressive maturation of autonomic regulation during childhood, characterized by increasing parasympathetic dominance and more stable cardiovascular control [15,16,21,23,24,25]. While these maturational trends were statistically robust, their effect sizes were modest, suggesting that physiological development rather than pathological processes underlies these changes.

In unadjusted analyses, the QT interval showed significant positive correlations with multiple HRV indices, including SDNN and RMSSD. At first glance, these findings may suggest a link between autonomic modulation and ventricular repolarization dynamics. However, after accounting for heart rate, these associations were no longer observed. This finding is particularly important, as both HRV parameters and the QT interval are strongly influenced by heart rate. Previous studies have demonstrated that heart rate is a principal determinant of HRV parameters and that changes in heart rate inherently influence HRV indices, potentially confounding their physiological interpretation [17,26,27]. Therefore, the apparent relationship between HRV and QT likely reflects a shared dependence on heart rate rather than a direct electrophysiological interaction. This interpretation is further supported by partial correlation analyses, which similarly demonstrated the absence of independent associations after controlling for confounders.

The lack of association between HRV parameters and QTc further supports this interpretation, as QTc accounts for heart rate effects. QTc is designed to adjust for heart rate effects, and the absence of correlation with HRV indices suggests that once heart rate dependency is accounted for, the relationship between autonomic variability and repolarization duration becomes negligible. Our findings suggest that the observed association between HRV and QT interval in this cohort is largely explained by their shared dependence on heart rate rather than an independent physiological relationship.

In contrast to the QT interval findings, repolarization heterogeneity, as reflected by QT dispersion (QTd), showed a modest but persistent association with SDNN even after multivariable adjustment. SDNN reflects global autonomic variability over 24 h and captures both sympathetic and parasympathetic influences. The persistence of this association suggests that global autonomic modulation may have a modest independent influence on the spatial heterogeneity of ventricular repolarization. However, given the modest effect size and known methodological limitations of QTd, this finding should be interpreted cautiously.

Sex emerged as the only independent predictor of QTc, with females exhibiting higher QTc values than males. This finding is consistent with well-established sex-related differences in cardiac electrophysiology, likely influenced by hormonal and genetic factors, particularly during pubertal development [5,8,28,29]. In addition, males demonstrated higher HRV values across most parameters, suggesting sex-specific differences in autonomic tone. These observations underscore the importance of considering sex as a biological variable in pediatric electrophysiological studies.

The age-specific percentile data provided in this study represent an important contribution to pediatric cardiology. While previous studies have primarily focused on pathological conditions, such as obesity or metabolic syndrome [13,14,30,31], our findings provide a reference framework for healthy children. A recent large-scale meta-analysis including nearly 3000 healthy pediatric subjects has proposed age-stratified reference limits for HRV parameters derived from Holter recordings. While this study provides valuable normative benchmarks, it is based on aggregated data and does not evaluate the relationship between HRV and ventricular repolarization parameters at the individual level [18]. These normative values may facilitate the interpretation of HRV parameters in clinical settings and support early identification of deviations from physiological patterns. In contrast, our study not only provides age-stratified normative HRV data but also examines the interaction between autonomic modulation and ventricular repolarization, suggesting that the observed associations between HRV and the QT interval are largely explained by heart rate.

Several limitations should be acknowledged. First, although only children with normal findings after comprehensive cardiovascular evaluation were included, the study population was recruited from a pediatric cardiology outpatient clinic, which may limit the generalizability of the findings, and the cross-sectional design precludes causal inference. Second, despite adjustment for key confounders such as age, sex, and heart rate, other factors influencing HRV—such as physical activity, sleep patterns, and pubertal status—were not assessed. Third, the present findings have not undergone external validation in an independent pediatric cohort, and future validation studies would help confirm their reproducibility. Although multiple correlation analyses were performed, the primary conclusions of this study are based on multivariable regression models adjusted for key confounders, reducing the likelihood that the findings are driven by type I error. Finally, QT dispersion is a parameter with known methodological limitations, particularly in Holter-based analyses, and should be interpreted with caution. The moderate reproducibility of QTd measurements (ICC = 0.72) may also reflect known methodological limitations of this parameter and should be considered when interpreting these findings. In addition, advanced ventricular repolarization markers, including the Tpeak–Tend interval, T-wave morphology, and QT variability index, were not available for analysis. Future prospective studies incorporating these parameters may provide a more comprehensive assessment of ventricular repolarization in children.

In conclusion, our findings indicate that the apparent association between heart rate variability and the QT interval in healthy children appears to be largely explained by heart rate rather than an independent physiological relationship. A modest association between global HRV and repolarization heterogeneity may persist; however, its clinical relevance appears limited. These results underscore the importance of accounting for heart rate when interpreting autonomic and repolarization parameters and highlight a key methodological consideration for future electrocardiographic studies in pediatric populations.

## Figures and Tables

**Table 1:** Descriptive Statistics of HRV and Ventricular Repolarization Parameters.

Parameter	Mean ± SD	Median (IQR)	Minimum	Maximum
Age (years)	12.51 ± 3.55	13.00 (4.00)	5.00	18.00
Heart Rate (bpm)	82.17 ± 10.77	81.00 (16.00)	44.00	115.00
Ventricular Repolarization				
QT Interval (ms)	366.97 ± 21.58	366.00 (28.00)	302.00	429.00
QTc (ms)	400.63 ± 19.43	401.00 (28.00)	354.00	456.00
QTd (ms)	149.88 ± 39.52	148.00 (44.25)	31.00	266.00
QTcd (ms)	140.10 ± 39.88	135.00 (40.75)	25.00	270.00
Heart Rate Variability				
SDNN (ms)	149.71 ± 43.49	143.50 (54.75)	40.00	366.00
SDANN (ms)	132.45 ± 38.62	126.80 (48.20)	35.00	320.00
RMSSD (ms)	46.74 ± 15.02	46.00 (20.00)	10.00	90.00
pNN50 (%)	24.18 ± 12.45	22.40 (16.80)	2.00	68.00
LF (ms^2^)	927.85 ± 410.06	890.00 (616.40)	107.00	2270.70
HF (ms^2^)	907.51 ± 643.73	744.80 (819.33)	39.30	3416.20
LF/HF Ratio *	1.32 ± 0.66	1.15 (0.73)	0.07	4.56

* The discrepancy between mean and median values for certain variables indicates a non-normal distribution, and these variables were interpreted using non-parametric statistical methods.

**Table 2:** Age-Specific Percentiles for 24 h HRV Parameters.

Age Group	Parameter	5th	10th	25th	50th	75th	90th	95th
5–8 years	SDNN (ms)	84.2	91.5	104.8	118.5	135.4	152.1	164.8
	RMSSD (ms)	22.4	26.5	32.1	38.4	46.2	54.8	61.2
	LF (ms^2^)	385.4	452.1	582.4	712.5	884.2	1042.1	1152.4
	HF (ms^2^)	302.1	364.5	482.1	642.0	812.4	985.4	1124.5
	LF/HF Ratio	0.62	0.74	0.88	1.05	1.24	1.48	1.62
9–12 years	SDNN (ms)	98.5	106.4	122.1	138.4	158.2	178.4	192.5
	RMSSD (ms)	26.8	31.2	37.4	44.5	52.8	62.1	68.4
	LF (ms^2^)	482.1	554.2	684.5	842.1	1042.8	1242.1	1384.5
	HF (ms^2^)	364.2	425.1	554.2	724.0	924.5	1142.1	1284.5
	LF/HF Ratio	0.74	0.82	0.96	1.12	1.35	1.58	1.74
13–15 years	SDNN (ms)	112.4	121.5	138.4	156.2	178.4	202.1	218.4
	RMSSD (ms)	30.5	35.2	42.1	49.8	58.4	68.5	76.2
	LF (ms^2^)	554.2	642.1	784.5	982.4	1212.4	1452.1	1624.5
	HF (ms^2^)	412.4	482.1	642.1	842.0	1082.4	1342.1	1524.5
	LF/HF Ratio	0.82	0.94	1.08	1.24	1.48	1.72	1.88
16–18 years	SDNN (ms)	124.5	134.2	152.4	174.5	198.4	224.5	242.1
	RMSSD (ms)	34.2	39.1	46.5	55.2	65.4	78.2	86.4
	LF (ms^2^)	624.1	712.4	884.2	1124.5	1384.1	1652.4	1842.1
	HF (ms^2^)	462.1	542.1	712.4	942.0	1212.4	1512.4	1712.4
	LF/HF Ratio	0.92	1.05	1.22	1.42	1.68	1.94	2.12

Note: Data derived from the healthy pediatric cohort (N = 254). Significant maturational shifts were observed (*p* < 0.001).

**Table 3:** Correlation Matrix Between HRV and Ventricular Repolarization Parameters.

	SDNN	SDANN	RMSSD	pNN50	LF	HF	LF/HF
QT Interval	0.434 ***	0.412 ***	0.426 ***	0.405 ***	0.437 ***	0.367 ***	−0.151 *
QTc	−0.030	−0.015	0.029	0.018	−0.022	0.024	−0.058
QTd	0.269 ***	0.214 **	0.146 *	0.122 *	0.114	0.186 **	−0.116
QTcd	0.016	0.008	−0.039	−0.025	−0.016	−0.027	0.045

Note: Pearson or Spearman correlation coefficients. * *p* < 0.05, ** *p* < 0.01, *** *p* < 0.001.

## Data Availability

The datasets generated and/or analyzed during the current study are available from the corresponding author on reasonable request, in accordance with institutional policies and ethical regulations.
